# A national observation study of cancer incidence and mortality risks in type 2 diabetes compared to the background population over time

**DOI:** 10.1038/s41598-020-73668-y

**Published:** 2020-10-15

**Authors:** Hulda Hrund Bjornsdottir, Araz Rawshani, Aidin Rawshani, Stefan Franzén, Ann-Marie Svensson, Naveed Sattar, Soffia Gudbjörnsdottir

**Affiliations:** 1 Department of Health Sciences, Faculty of Medicine, The University of Iceland, Reykjavík, Iceland; 2 The Swedish National Diabetes Register, Västra Götalandsregionen, Gothenburg, Sweden; 3 The Department of Molecular and Clinical Medicine, Institute of Medicine, University of Gothenburg, Gothenburg, Sweden; 4 Health Metrics Unit, The Sahlgrenska Academy, University of Gothenburg, Gothenburg, Sweden; 5 Institute of Cardiovascular and Medical Sciences, BHF Glasgow Cardiovascular Research Centre, University of Glasgow, 126 University Place, Glasgow, G12 8TA UK

**Keywords:** Cancer, Obesity, Type 2 diabetes

## Abstract

We examined changing patterns in cancer incidence and deaths in diabetes compared to the background population. A total of 457,473 patients with type 2 diabetes, included between 1998 and 2014, were matched on age, sex, and county to five controls from the population. Incidence, trends in incidence and post-cancer mortality for cancer were estimated with Cox regression and standardised incidence rates. Causes of death were estimated using logistic regression. Relative importance of risk factors was estimated using Heller’s relative importance model. Type 2 diabetes had a higher risk for all cancer, HR 1.10 (95% CI 1.09–1.12), with highest HRs for liver (3.31), pancreas (2.19) and uterine cancer (1.78). There were lesser increases in risk for breast (1.05) and colorectal cancers (1.20). Type 2 diabetes patients experienced a higher HR 1.23 (1.21–1.25) of overall post-cancer mortality and mortality from prostate, breast, and colorectal cancers. By the year 2030 cancer could become the most common cause of death in type 2 diabetes. Persons with type 2 diabetes are at greater risk of developing cancer and lower chance of surviving it. Notably, hazards for specific cancers (e.g. liver, pancreas) in type 2 patients cannot be explained by obesity alone.

## Introduction

Coronary heart disease (CHD) rates are half of what they were in the 1980s^1^ and data shows that cancer has now become the leading cause of death in many European countries^1–3^. A growing body of evidence demonstrates that type 2 diabetes confers an increased risk of total cancer and site-specific cancers and furthermore, that it may affect prognosis^4–13^. Type 2 diabetes and cancer share certain risk factors that might contribute to these associations (e.g. obesity, smoking, and diet^6,13,14^). That noted, existing evidence suffers to differing extents from biases such as residual confounding, investigation bias, uncertainty due to self-reported information, and reverse causality^5,6^. There is an abundance of studies evaluating the association of type 2 diabetes with cancer mortality^7,10,15–20^, but fewer studies on risk for incident cancers in type 2 diabetes in relation to matched controls^6^. Also, few studies have looked at both incidence and mortality in nationally representative populations. There is a need for a study with a large sample size examining both incidence and mortality from cancer for persons with type 2 diabetes. With cancer now being a more common cause of death and both incidence and prevalence of diabetes increasing worldwide^3,21,22^, the association between these two outcomes might make the burden of these diseases a substantial global health challenge.

We evaluated (1) time trends in causes of death, (2) incidence of all cancer and site-specific cancers, (3) time-trends in cancer incidence and (4) post-cancer mortality among patients with type 2 diabetes compared to matched controls from the general population. We also compared our incident cancer risks with previously published work on BMI and cancer^23^ in order to evaluate if type 2 diabetes may have an effect on risk for some cancers independent of obesity. Finally, we evaluated the ability of routine risk factors to predict cancer incidence for type 2 diabetes, using one of the world’s largest diabetes databases.

## Methods

### Study design and support

The study was supported by the European Foundation for the Study of Diabetes, the Swedish Association of Local Authorities and Regions and the Swedish Heart and Lung Foundation and all methods were performed in accordance with the relevant guidelines and regulations; no support was provided by industries. The ethics review board at the University of Gothenburg approved the study.

### Data sources

The Swedish National Diabetes Register (NDR), initiated in 1996, has been described previously^24,25^. The register contains information on risk factors, treatment and complications of diabetes. Each patient provides informed consent for inclusion in the database. In 2017, all specialist clinics participated and 95% of primary health care centers, and the register included approximately 90% of the diabetes population. For this study, persons with at least one listing in the NDR between 1998 and 2012 were included. Type 2 diabetes was defined by the epidemiologic definition: “Patients of all ages receiving only dietary treatment, or oral glucose-lowering agents only, or persons diagnosed after the age of 40 years receiving insulin therapy or insulin and oral glucose-lowering agents”. This definition has been validated against the clinician’s assessment of diabetes type, which concur in 96% of cases with no temporal differences. In particular, none had gestational diabetes, pre-diabetes or, as far as we can determine type 1 diabetes. For each individual with type 2 diabetes, five controls were randomly selected from the general population. The control group was retrieved from the total population register and was matched according to age, sex, and county. The controls are persons who during the study period do not develop T2DM and they enter into the cohort at the date when the corresponding T2DM person does. The cohort included 457,473 persons with type 2 diabetes and 2,2877,365 matched controls. The follow-up for persons with T2DM starts at the date of cohort entry.

The Total Population Register contains data on life events including birth, death, change of name, marital status and migration. Almost 100% of births and deaths, 95% of immigrations and 91% of emigrations are reported to the register within 30 days^26^. From this register we obtained our matched controls. Data on cancer outcomes and all-cause death were retrieved from the Swedish Cancer Registry and the Swedish Cause of Death Registry. Information on comorbidities was retrieved from Swedish Patient Register and socioeconomic variables were retrieved from the Longitudinal Integration Database for Health Insurance and Labour Market Studies (LISA register). The Swedish Cancer Register registers cancer diagnoses in Sweden since 1958. Every clinician, pathologist and cytologist are required to notify the register of every person who receives a diagnosis of a new primary malignancy. The register includes all primary malignancies and certain benign neoplasms and precancerous lesions^27^. The Swedish Cause of Death register contains data for cause of death from the year 1952. The Cause of Death register includes everyone who died during one calendar year that was registered in Sweden at the time of death, regardless of whether they died in Sweden or outside the country^28^. The LISA register is made from annual registers since 1990 and includes all persons 16 years and older who are registered in Sweden at December 31st each year. The LISA register incorporates existing data from registers on labor market, education and social districts using background information from population registers^29^.

Linkage of registries is virtually complete due to the use of unique personal identification numbers in Sweden, which are assigned to all habitants at birth or at the time of immigration.

### Outcomes

All individuals were followed for a site-specific cancer occurrence, death, or end of follow-up, whichever came first. Follow-up was until 2013 for cancer occurrences and 2014 for mortality. Date and type of cancer diagnosis was retrieved from The Swedish Cancer registry, using ICD-7 codes. The specific codes are listed in Supplementary Table 1 in the online-only supplementary material. Causes of death were retrieved from the Cause of Death Register, using ICD-10 codes. Cause of death was defined as the principal cause in the death certificates; causes were categorised according to their ICD categories, with cardiovascular death being ICD codes starting with I, and cancer being ICD codes starting with C and D. Yearly incidence rates were standardised by age and sex.

### Statistical analysis

Cause of death was classified as cancer related, cardiovascular or other and modelled as a function of calendar year using multinomial regression models with year as a continuous independent variable. The parameter estimates from the multinomial models were used for a rough prediction of when cancer and cardiovascular causes will be equally common.

We studied incidence of cancer and therefore patients with a pre-existing cancer diagnosis (in the Swedish Cancer Registry) for a certain site-specific cancer were excluded from that specific cancer in the incidence analysis. Incidence rates, expressed as the number of events per 10,000 person-years, were calculated for each outcome. To compare the incidence rates for type 2 diabetes persons versus controls, we constructed a Cox regression model for all cancer incidence and for each site-specific cancer incidence. In this model, participants were followed from the index date until an event (date of cancer diagnosis), or appropriate censoring, using age as the time scale. Models were adjusted for sex, education, income, and marital status. Even if the persons with T2DM and the controls are matched on age, sex and county we had both variables in the model to avoid the omitted variable bias that is a consequence of omitting important risk predictors from a Cox regression model. We repeated such analyses using patients with type 2 diabetes with less than 1 year diagnosis to relevant controls. We did this to minimise people with longer duration who may have higher cumulative cancer risk; useful therefore to also examine risks in recently (i.e. newly) diagnosed patients. To gain reasonable numbers, we chose to look at cancer risks in patients with less than 1 year of diabetes duration. The analysis was repeated as three separate landmark analyses where the first one, two and three years were excluded to investigate the possible effect of detection bias or reverse causality that might be present shortly after the onset of T2DM.

To further explore how the incidence rate develops as a function of diabetes duration in newly diagnosed T2DM we estimated the yearly incidence rate, with exact Poisson 95% confidence interval for each year following disease onset.

We also investigated the secular time trends in incidence for site specific cancers for type 2 diabetes versus controls by computing a Cox regression model with biannually time-updated calendar time as a continuous variable. The model also includes an interaction term between calendar time and diabetes status (type 2 diabetes or control) allowing separate effects of calendar time to be estimated for type 2 diabetes and controls. The parameters estimating the separate effect of calendar time for type 2 diabetes and controls were transformed by raising this coefficient to the fifth power to represent the ratio between type 2 diabetes and controls over a 10-year period. A ratio of those hazard ratios shows the relative difference between the groups.

Post-cancer mortality for type 2 diabetes versus controls was estimated with a Cox regression model. In this model we adjusted for sex and the last pre cancer observation on age, education, marital status, and income since among the persons diagnosed for cancer during the study we no longer have balance with respect to age and gender between T2DM and controls. This estimation describes all-cause mortality following a cancer diagnosis. Critically, we also examined cancer specific mortality given that all-cause mortality might capture higher deaths rates from type 2 diabetes in general.

In all comparisons between type 2 diabetes and controls an estimated hazard ratio above 1.0 was interpreted as a greater event-rate among persons with type 2 diabetes than among controls.

To evaluate our current ability to predict cancer occurrences we evaluated the relative importance of all risk factors available in our database using Heller’s relative importance model^30^. Heller's R^2^ generalises the normal R^2^ to a time-to-event setting. When estimating the influence of the risk factors we only included cancer-outcomes that were reported more than three years after the index date in an effort to mitigate reverse causality. In order to capture non linear associations between continuous risk factors and the outcomes studied, we modelled continuous variables using smoothing splines with five degrees of freedom. Smoothing splines essentially puts one knot at each observation and use penalization based on the total curvature in each knot (i.e. sum of the second derivatives) to allow the function to bend where it has to. The tuning parameter, the degrees of freedom, was set to five based on visual inspection of the spline curves and seems to capture most of the relevant variability without excessive wiggling. In an effort to both maximize the sample size and to avoid the assumption of missing completely at random we imputed missing data for the relative importance model using a single imputation using chained equations (MICE). Single imputation was chosen over multiple imputation due to the massive computational time.

A *p* value of < 0.05 was considered to indicate statistical significance, without any adjustment for multiple comparisons, implying that interpretations need to be cautious and based on patterns rather than single hypothesis tests. All statistical analysis was conducted with SAS (version 9.4) and Rstudio (version 3.4.3).

## Results

### Baseline characteristics

Table 1 shows baseline characteristics in the overall cohort as well as broken down by diabetes diagnosed with less than 1 year of follow-up. Baseline characteristics were defined at the type 2 diabetes person’s first observation in the NDR, which was also the index date for the matched controls. For the entire cohort, the mean age in both groups was 65.2 years. Median follow-up was 6.6 years. As could be expected, histories of cardiovascular diseases (CVD), and related outcomes were more frequent among persons with type 2 diabetes. The mean age of those diagnosed with less than 1 year of follow-up was around 63.2 years and fewer diabetes patients had prior CVD or related complications but patterns of difference were broadly similar. The mean duration was 0.4 months in this case. As seen in Supplementary Table 2, the proportion of missing data ranged from practically zero for age and sex to very high (53.8%) for physical activity. Key variables such as HbA1c and BMI had 10.7% and 24.7% missing, respectively.

**Table 1:** Baseline characteristics for type 2 diabetes and controls using the entire cohort and restricting to those with less than one year since diagnosis.

Variable	Type 2 diabetes (n = 457,473)	Controls (n = 2,287,365)	Type 2 diabetes < 1 year (n = 208,049)	Controls (n = 1,040,245)
Age—mean years (SD)	65.2 (12.6)	65.2 (12.6)	63.2 (12.9)	63.2 (12.9)
Female—no (%)	208,019 (45.47%)	1,040,095 (45.47%)	95,224 (45.77%)	476,120 (45.77%)
**Place of birth—no (%)**				
Sweden	375,703 (82.13%)	2,003,918 (87.61%)	168,095 (80.80%)	902,774 (86.78%)
Europe, outside Sweden	48,676 (10.64%)	207,022 (9.05%)	22,747 (10.93%)	95,997 (9.23%)
Rest of the world	33,094 (7.23%)	76,425 (3.34%)	17,207 (8.27%)	41,474 (3.99%)
**Education—no (%)**				
College level education	179,368 (40.16%)	882,155 (39.27%)	87,120 (42.74%)	421,488 (41.15%)
Elementary school education	196,206 (43.93%)	829,593 (36.93%)	81,095 (39.78%)	336,101 (32.81%)
Upper secondary school education	71,028 (15.90%)	534,677 (23.8%)	35,632 (17.5%)	266,797 (26.0%)
**Coexisting conditions—no (%)**				
CHD	79,096 (17.29%)	200,912 (8.78%)	30,536 (14.68%)	79,440 (7.64%)
HF	30,686 (6.71%)	74,807 (3.27%)	11,514 (5.53%)	28,376 (2.73%)
Hyperglycemia	5976 (1.31%)	704 (0.03%)	1360 (0.65%)	175 (0.02%)
Amputation	1729 (0.38%)	2368 (0.10%)	472 (0.23%)	958 (0.09%)
Psychiatric disorders	13,651 (2.98%)	44,934 (1.96%)	7288 (3.50%)	21,816 (2.10%)
Renal disorders	1021 (0.22%)	3004 (0.13%)	350 (0.17%)	1268 (0.12%)
Cancer	33,799 (7.39%)	161,217 (7.05%)	14,560 (7.00%)	69,292 (6.66%)
Gastric bypass	322 (0.07%)	602 (0.03%)	115 (0.06%)	343 (0.03%)
Diabetes duration—year (SD)	5.7 (7.1)		0.4 (0.5)	
BMI (kg/m^2^)—mean (SD)	29.7 (5.4)		30.3 (5.6)	
HbA1c(mmol/mol): median (Iq)	54.5 (14.9)		52.8 (15.7)	
SBP(mmHg)—mean (SD)	140.2 (18.3)		138.2 (17.8)	
DBP(mmHg)—mean (SD)	78.7 (9.9)		79.4 (10.0)	
Total Chol (mmol/L)—mean (SD)	5.1 (1.1)		5.2 (1.1)	
LDL(mmol/L)—mean (SD)	2.9 (1.0)		3.1 (1.0)	
HDL(mmol/L)—mean (SD)	1.3 (0.4)		1.2 (0.4)	
Trig(mmol/L)—median (iq)	1.9 (1.2)		2.0 (1.3)	
eGFR—median (Iq)	80.5 (25.3)		83.2 (25.1)	
**Diabetes treatment—no (%)**				
Diet only	172,642 (37.7%)		108,655 (52.2%)	
Tablets	195,194 (42.6%)		82,496 (39.6%)	
Insulin	47,635 (10.41%)		10,312 (4.96%)	
Tablets and insulin	42,246 (9.23%)		6586 (3.17%)	
Anti-hypertensive treatment	276,011 (64.3%)		119,017 (61.6%)	
Lipid lowering treatment	170,342 (39.8%)		72,491 (37.5%)	
Micro Albuminurea	22,009 (6.89%)		6215 (4.81%)	
Macro Albuminurea	39,570 (15.43%)		13,502 (12.87%)	
Smoking	57,458 (15.62%)		27,424 (17.46%)	

*CHD* coronary heart disease, *CVD* cardiovascular disease, *AMI* acute myocardial infarction, *AF* atrial fibrillation, *HF* heart failure, *BMI* body mass index, *BP* blood pressure, *eGFR* estimated glomerular filtration rate, *HbA1c* glycated hemoglobin, *HDL* high density lipoprotein, *LDL* low density lipoprotein, *n* number of patients.

Diagnostic codes for the conditions listed are from the *International Classification of Diseases, 9th Revision* and *10th Revision*.

Percentages for the glycated hemoglobin level were based on values from the National Glycohemoglobin Standardization Program.

The body-mass index is the weight in kilograms divided by the square of the height in meters.

The glomerular filtration rate (GFR) was estimated with the use of the Modification of Diet in Renal Disease equation.

Concentrations of glycated hemoglobin were based on values from the International Federation of Clinical Chemistry and Laboratory Medicine.

### Causes of death

**Figure 1:**
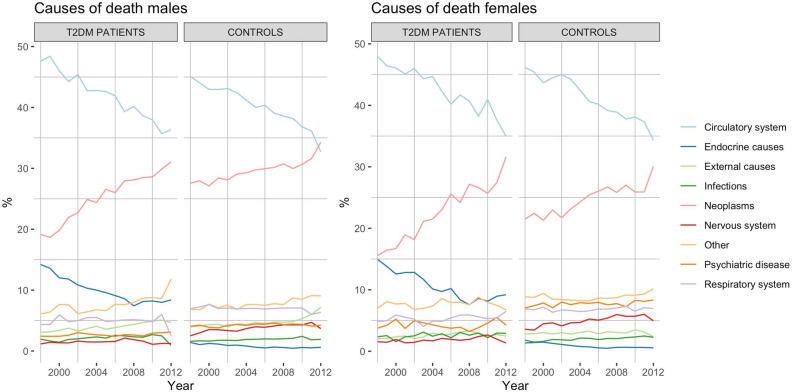
Causes of death in Sweden between 1998 and 2012. (**a**) Results for males with type 2 diabetes and controls, (**b**) results for females with type 2 diabetes and control.

Figure 1 presents causes of death in patients with type 2 diabetes and controls from year 1998 to 2012. Patients with type 2 diabetes primarily died from cardiovascular and endocrine causes in the initial years. However, rates of cardiovascular and endocrine death abated each year, such that CVD and endocrine causes represented 62.3% of deaths in the first year, and 44.4% of deaths in the final year. By contrast, cancer mortality increased gradually. In relative numbers, female cancer death rates increased by 103% with type 2 diabetes and 40% for the matched controls. Death from cancer increased by 62% for males with type 2 diabetes while increasing only by 24% for matched controls. Remaining causes of death did not display any material trend over time. The logistical regression model suggested, if current trends continue, that by the year 2030 cancer would become the most common cause of death among individuals with type 2 diabetes and by 2040 in controls.

### Cancer incidence and mortality

#### Risks in diabetes for the most common sites of cancer

**Figure 2:**
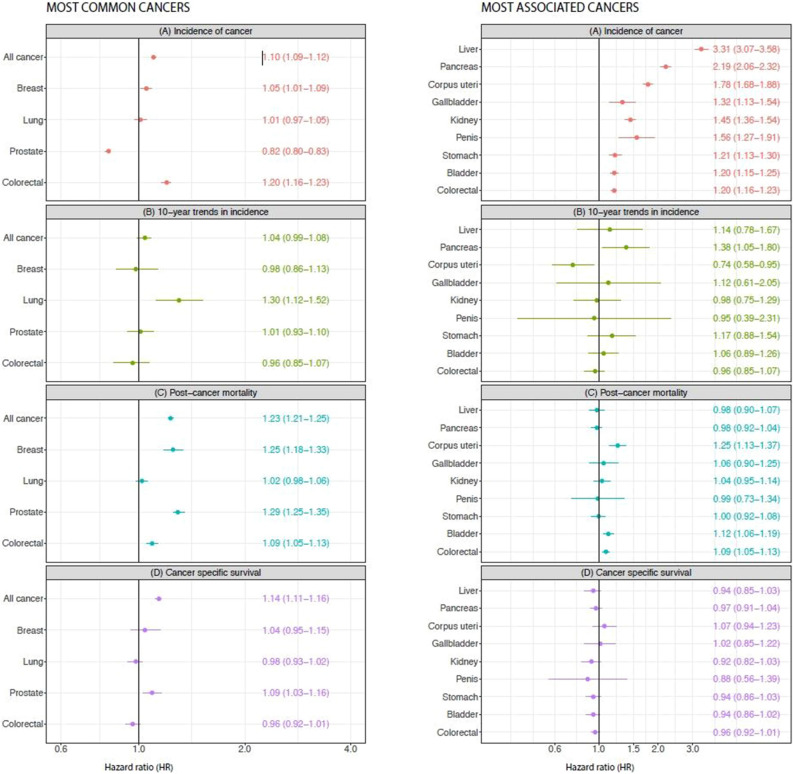
Hazard ratios for cancer incidence, 10-year trends, post-cancer mortality, and cancer specific survival in Type 2 diabetes patients versus matched controls. (Red) Hazard ratios of incidence; (Green) hazard ratios for 10-year trends in incidence; (Blue) hazard ratios for post-cancer mortality for the most common (**a**) and most associated (**b**) cancer sites. Incidence model adjusted for sex, education, income, marital status and geographical region of birth. Time trends in incidence were adjusted for age and gender. Post-cancer mortality model adjusted for sex, age, education, marital status and income.

Figure 2a displays results for the most common cancer sites in our cohort, which are in line with worldwide incidence rates of cancer. Results for all specific cancer sites can be found in the online-only supplementary material (Supplementary Figure 1).

Figure 2a panel A shows hazard ratios of incidence for all cancer and the four most common cancer sites. For all cancer, type 2 diabetes had a higher risk, with a HR of 1.10 (95% CI 1.09–1.12). For the most common cancer sites, risks HR were above one at 1.05 (95% CI 1.01–1.09) for breast cancer and 1.20 (95% CI 1.16–1.23) for colorectal cancer. They were lower for prostate cancer, and no different for lung cancer.

Table 2 displays a comparison of cancer rates when we compared the entire cohort to risks in those with less than 1 year of follow-up relative to matched controls in each case. Of importance, the patterns of cancer risk were broadly same, though notably, overall cancer risks were higher at 1.22 (1.17–1.27) and risks for most individual cancers were somewhat higher, notably for pancreatic cancer with HR of 2.72. However, in nearly all cases, HRs overlapped.

**Table 2:** Hazard ratios for cancer incidence using the entire cohort and then restricting diabetes patients to those with < 1 year since diagnosis (mean duration 4 months). HRs are relative to age, sex and county-matched controls in each case, as per data in Table 1.

	All diabetes	Newly diagnosed (< 1 year)	
**Most common cancers**			
All cancer	1.10 (1.09–1.12)	All cancer	1.22 (1.17–1.27)
Breast	1.05 (1.01–1.09)	Breast	1.12 (1.02–1.23)
Lung	1.01 (0.97–1.05)	Lung	1.18 (1.04–1.34)
Prostate	0.82 (0.80–0.83)	Prostate	0.80 (0.70–0.90)
Colorectal	1.20 (1.16–1.23)	Colorectal	1.30 (1.18–1.43)
**Most associated cancers**			
Liver	3.31 (3.07–3.58)	Liver	2.36 (1.74–3.20)
Pancreas	2.19 (2.06–2.32)	Pancreas	2.72 (2.28–3.25)
Corpus uteri	1.78 (1.68–1.88)	Corpus uteri	1.68 (1.44–1.96)
Gallbladder	1.32 (1.13–1.54)	Gallbladder	1.55 (1.02–2.37)
Kidney	1.45 (1.36–1.54)	Kidney	1.78 (1.45–2.19)
Penis	1.56 (1.27–1.91)	Penis	1.96 (0.77–4.99)
Stomach	1.21 (1.13–1.30)	Stomach	1.37 (1.06–1.77)
Bladder	1.20 (1.15–1.25)	Bladder	1.15 (0.96–1.37)
Colorectal	1.20 (1.16–1.23)	Colorectal	1.30 (1.18–1.43)

Model adjusted for sex, education, income and marital status.

Figure 2a panel C displays hazard ratios of *post-cancer mortality* for the most common cancer sites in type 2 diabetes as compared to controls. For overall cancer mortality we observed a HR of 1.23 (95% CI 1.21–1.25) for patients with type 2 diabetes, compared with controls. They were also higher for breast, colorectal and prostate cancer but not for lung cancer.

Figure 2a panel D displays hazard ratios of *post-cancer cancer-specific mortality.* Overall, we observed a HR of 1.14 (95% CI 1.11, 1.16) for patients with type 2 diabetes, compared with controls, with higher cancer-specific mortality seen in prostate, HR 1.09 (95% CI 1.03–1.16), but not for breast or colorectal cancer.

#### The sites most associated with higher risk of cancer in patients with type 2 diabetes

Figure 2b panel A shows hazard ratios of incidence for the most associated cancer sites for type 2 diabetes as compared to controls, which were: liver, pancreas, uterus, penis, kidney, gallbladder and bile ducts, stomach and bladder, along with colorectal cancer. We observed the highest hazard ratios for liver cancer HR 3.31 (95% CI 3.07–3.58); pancreas cancer HR 2.19 (95% CI 2.06–2.32); and corpus uterus cancer HR 1.78 (95% CI 1.68–1.88). Overall, the excess risk of these cancers ranged from 1.2- to 3.3-fold.

Figure 2b panel C shows hazard ratios for post-cancer mortality for sites most associated with type 2 diabetes as compared to controls. Overall mortality risks were higher in type 2 diabetes only for uterine and bladder cancer.

Figure 2b panel D displays hazard ratios of *post-cancer and cancer-specific mortality* for the most common cancer sites. There was no real evidence of meaningful excess cancer-specific mortality for any of the cancers which are most associated with type 2 diabetes.

### Trends in cancer incidence

**Figure 3:**
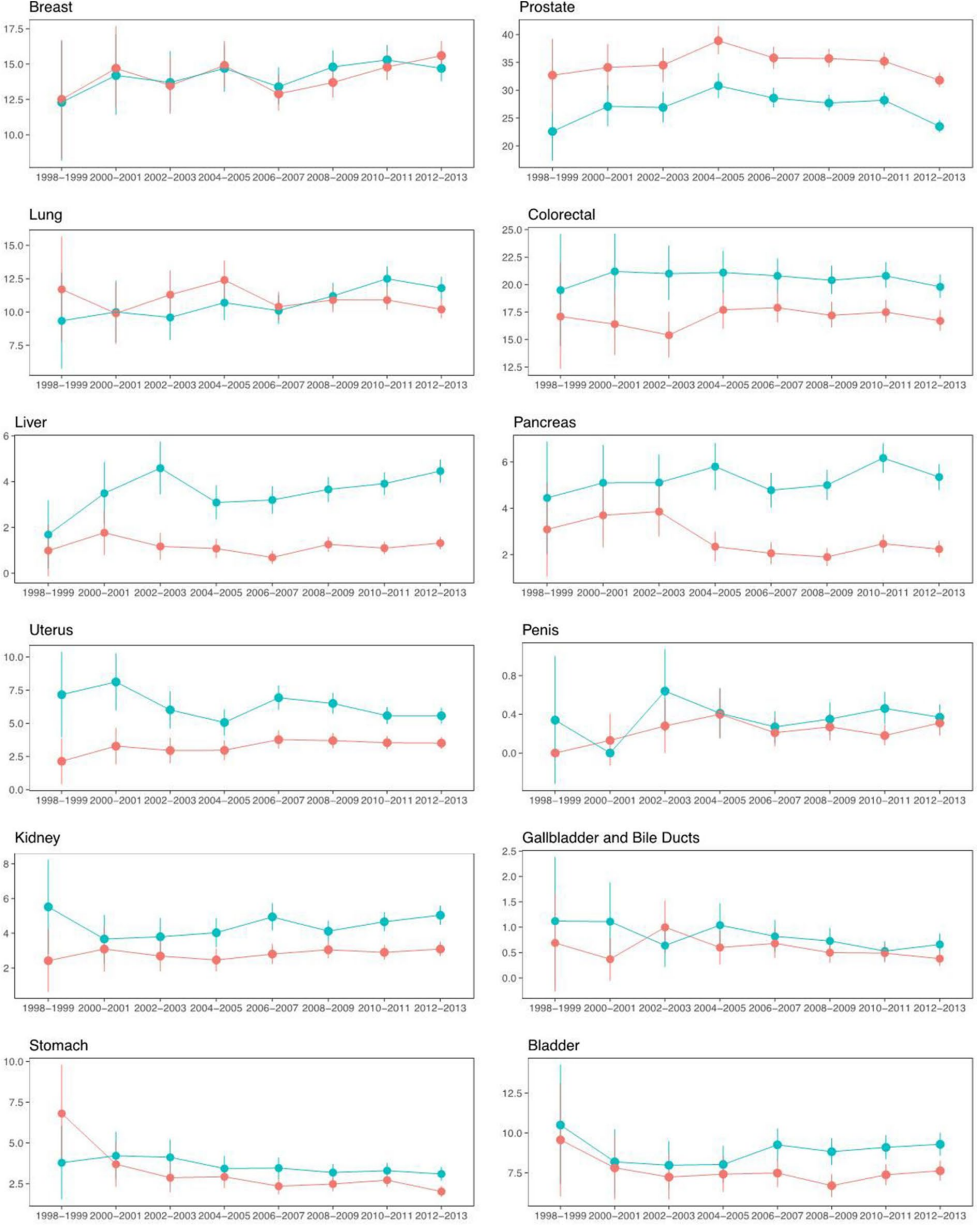
Time trends in cancer incidence. The most common and most associated sites. The x-axes show calendar years from 1998 to 2013. The y-axes display standardised incidence rates (note the differing limits on the y-axes). Blue lines represent people with type 2 diabetes and red lines represent matched controls from the general population. Model was adjusted for sex, education, income and marital status.

Figure 3 along with panels B of Fig. 2 displays time trends in cancer incidence. Of the four most common cancer sites a significant difference in change of risk over time was only observed for lung cancer, with a 30% greater increase in incidence over a 10-year period for type 2 diabetes compared to controls. For the sites that were most associated with type 2 diabetes, only pancreatic cancer and uterine cancer showed a significant difference in change of risk over time in type 2 diabetes as compared to controls, with a 38% greater increase in incidence over a 10-year period for pancreas cancer, but a 26% greater decrease for uterine cancer for type 2 diabetes compared to controls.

### Risk factor analysis

Supplementary Figure 2 shows relative importance of traditional risk factors collected in routine diabetes practice. All 15 risk factors collectively contributed very little to overall cancer prediction.

When we looked at relative importance separately by sex and in non-smokers only (Supplementary Figures 3 and 4) we noticed BMI to be the most important risk factor for cancer in women (R^2^ = 0.0042) but not in men (R^2^ = 0.00012) in whom eGFR (R^2^ = 0.0010) and physical activity (R^2^ = 0.00049) showed greatest relative importance, although, once again, overall risk factors contributed little to cancer prediction. Notably, when we added diabetes duration, this did not make a meaningful difference to associations (Supplementary Figure 5–7).

Finally, to further address detection bias and reverse causation, we added yearly incidence rates following type 2 diabetes diagnosis (split by sex) where we found that only pancreas cancer had a higher incidence rate during the first years following diagnosis of type 2 diabetes (Supplementary Figure 8). For interest, we also present the data for all cancers (Supplementary Figure 9). We have also performed landmark analyses comparing type 2 diabetes to controls, where we exclude the first one, two and three years of follow up (Supplementary Table 3). These data show nearly all associations remain robust with little variance, perhaps excluding pancreas cancer where we see a lowering of overall HR for all cancers from 1.10 (1.09, 1.12) to HR 1.07 (1.05, 1.08), the latter being the 3 year landmark analyses. That noted, some associations became stronger (specifically liver) whereas others become modestly weaker (e.g. Pancreas and stomach), though most HRs seemed to remain broadly consistent.

## Discussion

Even though general cancer trends in Sweden seem to match those in other high income countries^31^, this is one of the largest studies to present several dimensions in cancer-associated risks in type 2 diabetes. We demonstrate a 10% higher risk of cancer in general in those with type 2 diabetes (22% higher if we restricted analyses to diabetes patients with less than 1 year of duration), with risks even higher in several cancers linked to obesity (uterine, colorectal and breast). We furthermore present a 14% overall higher cancer-specific mortality and a rapid shift towards greater relative cancer mortality: women with type 2 diabetes experienced a 103% increase in cancer death rates, whereas men experienced a 62% increase. Corresponding figures for people without type 2 diabetes were 40% and 24%, respectively. Consequently, if current trends continue, by the year 2030 cancer may become the most common cause of death in type 2 diabetes and by 2035 in controls; yet, our ability to predict cancer is at best poor. This suggests it may take longer for cancer mortality to equal CVD mortality in patients with diabetes since PURE data recently showed cancer deaths to have already overtaken CVD deaths in general in middle age people in high income countries^32^.

Moreover, by comparing our incident risks with prior excellent research linking BMI to cancers^23^ we clearly show that type 2 diabetes has an effect on the risk for many cancers (in particular liver and pancreas) that goes well beyond any effect of total adiposity per se. Our results in Sweden for overall cancer incidence risks, and observed magnitudes and patterns of risk by cancer type, are very much in line with those reported by Harding et al^33^ in 2015 who examined standardised incidence ratios in over 800 K people with type 2 diabetes in Australia, albeit over a shorter 11 years time period. Our findings therefore have strong external validity. Furthermore, whilst both Harding et al. and our study in Sweden showed pancreatic cancer to have the strongest evidence of detection bias, in both cases the level of excess risk remained high after the first few months or years. Whilst we could not see any great evidence of detection bias for other types of cancer, Harding et al., whose study had more power, did note some minor evidence for such bias for some cancers. However, once again, such potential biases were modest and insufficient to explain excess risks for many cancers. The two studies together therefore provide strong evidence for excess cancer risks in type 2 diabetes that cannot be explained by detection bias.

The individual cancer data were broadly comparable to findings in the entire cohort when we used patients with less than one years duration although, due to smaller numbers, the confidence intervals were inevitably wider (Table 2). Notably, however, the point estimate of risk for total cancers was not lower in this group but, if anything, it was slightly higher (1.22 vs 1.10). That said, both approaches have different potential biases but the fact that each gave similar patterns in risks is reassuring.

We believe our data show that obesity cannot explain all the excess risks of cancers in type 2 diabetes. The excess risks were markedly pronounced for liver (221% higher risk or 136% higher for newly diagnosed diabetes) and pancreatic (119% higher risk or 176% higher for newly diagnosed diabetes) cancers. These risk levels are far greater (by around five–tenfold) than Bhaskaran et al. previously^23^ suggests could be accounted for by obesity which showed that a five unit higher BMI (a typical type 2 diabetes to non-type 2 diabetes cohort BMI difference), was associated with an increased liver cancer risk by only 26% and pancreatic by 11% in non-smokers (Supplementary Table 4). Our work therefore extends Pearson-Stuttard et al.’s^8^ observations suggesting independent effects of obesity and diabetes on cancers, with greater type 2 diabetes effects on cancers like liver and pancreas. Thus, pathways leading to glucose dysregulation must add considerable neoplastic potential for these two sites. In addition, risks for several non-obesity related cancers (bladder by 20%, penile by 56%, and lip by 27%) were also higher in type 2 diabetes, whereas risks for other cancers (e.g. stomach [20%], colorectal [20%], kidney [45%]) also seem greater than a 5-unit higher BMI would predict^23^. These observations collectively suggest that type 2 diabetes-associated excess cancer risks, whilst contributed to by obesity, simply cannot be explained by this risk factor alone and that some other aspects of diabetes pathogenesis or hyperglycaemia must be involved. The fact that cancer risks are also increased in type 1 diabetes, adds further support for a potential role of hyperglycaemia^34^.

Perhaps most importantly, after a diagnosis of cancer, patients with type 2 diabetes had a 14% higher risk of death due to cancer. These findings suggest any bias in cancer detection in type 2 diabetes is at best modest, and that once cancer develops, greater overall mortality is evident compared to non-diabetes people with cancer. Notably, whilst post cancer mortality was no different for many aggressive cancers (e.g. liver, pancreas, stomach etc.), it was 9% (95% CI 3–16%) higher after a diagnosis of prostate cancer, suggesting either later detection in type 2 diabetes, perhaps due to obesity-related effects on PSA levels, or that such cancers may be more aggressive^35^.

### Cancer incidence and time trends in incidence

With the most common cancer sites accounting for four out of ten cancer occurrences worldwide^36^, even the small increase or decrease in incidence for type 2 diabetes noted in our study might have a major impact. Patients with type 2 diabetes displayed a 30% (95% CI 1.12–1.52) greater increase in lung cancer incidence over the study period which is interesting and perhaps unexpected. These results might be explained by competing risk. With mortality from cardiovascular disease decreasing for type 2 diabetes compared to controls^37,38^, patients with type 2 diabetes are surviving their cardiovascular diseases, and hence living longer, and as a consequence lung cancer incidence increases in this group. Smoking is of course a major risk factor of lung cancer and is associated with both type 2 diabetes and CVD^10^. Interestingly when analysis was made separately for males and females for lung cancer, we found that for females there was an increasing incidence rate for both T2DM and controls, while for men the incidence rate was constant for persons with T2DM and decreasing for the controls. That noted, when we did a formal competing risk analysis (by fitting a Fine & Grey model including lung cancer and death from any cause as the two competing events), the estimated subdistribution hazard ratio for lung cancer comparing persons with T2DM to controls turned out to be 1.03 with 95% CI (0.99, 1.06) which failed to reach statistical significance with *p* = 0.1731. For uterine cancer, there was a 78% higher risk for T2DM compared to controls but a 26% greater decrease in this excess risk over a 10-year period in type 2 diabetes. Long-term incidence trends also revealed that type 2 diabetes had a 38% greater increase in risk of pancreatic cancer over time. The reasons for these changes are not clear, and more work is needed to better understand and validate pancreatic risk changes over time in type 2 diabetes. If such risks are increasing, as we seem to show, this is a worrying trend given mortality rates from pancreatic cancer remain very high.

### Post-cancer mortality

The paradoxical finding, that the incidence of prostate cancer is lower, but mortality in total is higher in type 2 diabetes as compared to controls, has been described previously^5,10,39–42^. Cancer specific mortality was higher by 14% for all cancers when combined and, interestingly, significantly elevated only in prostate cancer, a novel finding. The sites that did not show a significant difference between groups for post-cancer mortality (or indeed cancer-specific mortality) were lung, gallbladder and bile ducts, kidney, stomach, liver, and pancreas. For all but penis cancer these results stem from the fact that these are generally aggressive cancers and they have a low average 5 year survival^43^.

### Strengths and limitations

We acknowledge we only had risk factor data, including BMI and smoking, in type 2 diabetes patients and whilst the patterns of excess cancer risks somewhat match those previously linked to obesity per se, it is clear that risks for some cancers are far in excess of any possible links to obesity whereas for others risks are higher than that explainable by obesity alone. In addition, risks are also increased for other cancers not previously linked to obesity. Of the cancer sites we found to be associated with T2DM, studies have shown that smoking is strongly associated with lung, stomach, kidney, bladder, pancreas, upper digestive tract and liver cancer^10^. It is also thought to be an independent risk factor for T2DM development, and it is therefore likely that T2DM cohorts smoked more than the controls^10^. In our cohort there were 15% who were reported as smokers and it is estimated that 9% of the Swedish general population smokes^44^. The relative importance model found smoking to be the second most important risk factor for the development of all cancer, but still only explaining very little of the variation in all cancer risk. It is therefore possible that our estimations of association between T2DM and cancer are somewhat confounded by smoking. We also did not have BMI in our control population and could therefore not examine the impact of undiagnosed cancer, as possibly evidenced by cachexia, on the risk of cancer in people with diabetes. Finally, we recognise the potential for detection bias and reverse causation could be relevant to our findings and to this end, we provide data to show the risks for key cancers we discuss remain intact even after removing the first three years of follow-up. We also show only pancreatic cancer seemed to be picked up more in the year after diagnosis, whereas little evidence for other cancers.

We did not include analysis on diabetes treatment in our study but do not consider that to be a limitation. The role of diabetes medication in cancer risk has been immensely studied^10,45–51^. There are numerous studies that show a decrease in cancer incidence for patients on metformin^4,10,11,47,51–53^. However, most of these findings have been found to be due to systematic bias such as immortal time bias, time-lag bias and time-window bias. This is partly due to misclassification of study participants, resulting in a reduction of risk. Many newer observational studies that minimized the effect of systematic bias did not find an association between metformin use and cancer risk reduction^47,52–54^. Metformin is the drug of choice for T2DM and results have been confounded by the fact that the drug is usually prescribed to people with a short duration of diabetes and those who do not have contraindicating factors that might be factors involved in risk of cancer^10,54,55^.

The ability to track both incidence and post-cancer total and cause-specific mortality helps mitigate concerns about potential biases from detection bias and reverse causation, although we accept some such biases remain possible. We realise that the prevalent cases may have suffered from a depletion of ‘susceptibles’. However, when we looked at the incident subcohort and plotted cumulative incidence as a function of time from diagnosis we found no evidence of any short-term increase in incidence rate during the first years, except for pancreatic cancer. We also examined risks in those with diabetes diagnosed under a year and found our results to be broadly similar to those in the fuller cohort. Even so, for sake of clarity, we have presented findings from both approaches so that readers can compare and contrast results. Finally, this work reflects predominately risks in white individuals in a high-income country and thus it cannot be used as a proxy for such work in low and middle-income countries where patterns of disease risks will be different due to rising cardiovascular risks.

## Conclusion

Persons with type 2 diabetes are at greater risk of developing cancer and a lower chance of surviving it, although such risks vary by cancer types. Whilst obesity might explain excess risks for many cancers, risks for specific cancers go well beyond obesity. As proportionately more people with type 2 diabetes are dying from cancer over time, cancer may become its most common cause of death within a decade if current trends continue, a pattern lagging behind such trends in the general population. Even so, more work on how to better prevent cancers in type 2 diabetes is urgently needed.

## Supplementary information

Supplementary Information.

## Data Availability

The datasets generated during and/or analysed during the current study are available from the corresponding author on reasonable request.
