# Supplementary material for: A national observation study of cancer incidence and mortality risks in type 2 diabetes compared to the background population over time
Source: Sci Rep. 2020 Oct 15;10:17376. doi: 10.1038/s41598-020-73668-y (PMC7566479; doi:10.1038/s41598-020-73668-y)

A national observation study of cancer incidence and mortality risks in type 2 diabetes compared to the background population over time

Hulda Hrund Bjornsdottir,[1,2] Araz Rawshani,[2,3] Aidin Rawshani,[2,3]
Stefan Franzén,[2,4] Ann-Marie Svensson,[2] Naveed Sattar,[5*] Soffia Gudbjörnsdottir,[2,3]

Affiliations:
[1)] The University of Iceland, Department of Health Sciences, Faculty of Medicine, Reykjavík, Iceland
[2)] The Swedish National Diabetes Register, Västra Götalandsregionen, Gothenburg, Sweden
[3)] The Department of Molecular and Clinical Medicine, Institute of Medicine, University of Gothenburg, Gothenburg, Sweden
[4)] Health Metrics Unit, the Sahlgrenska Academy, University of Gothenburg, Sweden
[5)] The Institute of Cardiovascular and Medical Sciences, University of Glasgow, Glasgow, United Kingdom

Corresponding author
Naveed Sattar
Institute of Cardiovascular & Medical Sciences, University of Glasgow, BHF Glasgow Cardiovascular Research Centre, 126 University Place, Glasgow, G12 8TA, UK
Email: naveed.sattar@glasgow.ac.uk
Tel: +44 (0)141 330 3419
ORCID iD: 0000-0002-1604-2593

Supplementary Material

Supplementary Table 1 The ICD codes, revision 7 and 10 and the names given to the variables in our analysis
Supplementary Table 2 The percentage of main characteristics with missing data
Supplementary Table 3 HR comparing diabetics to controls adjusted for socioecominic variables for main anlaysis and excluding the first 1, 2 and 3 years of follow up
Supplementary Table 4 Hazard ratios in current study for most strongly associated cancers in type 2 diabetes vs. Hazard ratios associated with 5 unit BMI difference shown study in a mainly white European cohort

Supplementary Figure 1 Results for all cancer sites
Supplementary Figure 2 Relative importance of risk factors (R2) estimated with the Heller model
Supplementary Figure 3 Relative importance of risk factors (R2) estimated with the Heller model in male non-smokers
Supplementary Figure 4 Relative importance of risk factors (R^2) estimated with the Heller model in female non-smokers
Supplementary Figure 5 Relative importance of risk factors (R2) estimated with the Heller model, incuding diabetes duration
Supplementary Figure 6 Relative importance of risk factors (R2) estimated with the Heller model in male non-smokers, incuding diabetes duration
Supplementary Figure 7 Relative importance of risk factors (R^2) estimated with the Heller model in female non-smokers, incuding diabetes duration
Supplementary Figure 8 Yearly incidence of pancreatic cancer per 10000 person years by time from diabetes diagnosis
Supplementary Figure 9 Yearly incidence of all cancers per 10000 person years by time from diabetes diagnosis

Supplementary Table 1 The ICD codes, revision 7 and 10 and the names given to the variables in our analysis

ICD-7	***ICD-10***	**Tumor Type**
140	*C00*	Lip
140-8	*C00-14*	Oral cavity all
141	*C01-02*	Tongue
143-4	*C03-06+C46.2*	Mouth
142	*C07-08*	Salivary
145-8	*C09-14*	Pharynx
150	*C15*	Esophagus
151	*C16*	Stomach
152	*C17*	Small intestine
153-4	*C18-21*	Colorectal
155.0	*C22*	Liver
155.1	*C23-24*	Gallbladder and bile ducts
157	*C25*	Pancreas
160	*C30-31*	Nose
161	*C32*	Larynx
162.0,1,8	*C33-34*	Lung
162.2	*C38.4+C45.0*	Pleura
196	*C40-41*	Bone
190	*C43*	Melanoma
197	*C49+C46.1*	Soft tissue
170	*C50*	Breast
171	*C53*	Cervix
172	*C54*	Corpus uterus
175	*C56,C57.0-4*	Ovary
179	*C60+C63*	Penis
177	*C61*	Prostate
178	*C62*	Testis
180	*C64*	Kidney
181	*C65-68+D09.0+D41.4*	Bladder
192	*C69*	Eye
193	*C70-72+D32-33+D42-43*	Brain
194	*C73*	Thyroid
201	*C81*	Hodgkin
200+202	*C82-85,C96*	Non-Hodgkin
203	*C90*	Multiple Myeloma
204	*C91-95*	Leukemia
204.0-2,8	*C91-95\C9X.0*	Other leukemia
204.3	*C91.0+C92.0+C93.0+C94.0+C95.0*	Acute leukemia
140-204\191	*CXX.X\(C44+C46.0)+D09.0+D41.4+D32-33+D42-43*	All cancer

Supplementary Table 2 The proportion of missing data for each characteristic

Variables	T2DM (n= 457473)	Percentage missing
**Age**	65.2 (12.6)	0.0
**Female**	208019 (45.47%)	0.0
**Age at diagnosis, year**	65.2 (12.6)	0.0
**Diabetes duration**	5.7 (7.1)	11.2
**BMI(k/m2)**	29.7 (5.4)	24.7
**HbA1c(mmol/mol)**	54.5 (14.9)	10.7
**SBP (mmHg)**	140.2 (18.3)	14.1
**DBP (mmHg)**	78.7 (9.9)	14.1
**Total Cholesterol(mmol/L)**	5.1 (1.1)	39.5
**LDL-c (mmol/L)**	2.9 (1.0)	48.5
**HDL-c (mmol/L)**	1.3 (0.4)	46.7
**Triglycerides(mmol/L)**	1.9 (1.2)	45.7
**Creatinine ()**	80.7 (30.0)	30.3
**eGFR**	80.5 (25.3)	30.3
**Diabetes treatment**	172543 (37.72%)	0.0
**Diet only**	195133 (42.65%)	
**Tablets**	47575 (10.40%)	
**Insulin**	42222 (9.23%)	
**Tablets and insulin**	275881 (64.29%)	
**Anti-hypertensive treatment**	170302 (39.83%)	6.2
**Lipid lowering treatment**	39552 (15.43%)	6.5
**Micro Albuminurea**	21967 (6.88%)	44.0
**Macro Albuminurea**	57437 (15.62%)	30.2
**Smoking**		19.6
**Physical Activity**		53.8
**Never**	31881 (15.07%)	
**<1 time/week**	27421 (12.96%)	
**1-2 times/week**	44689 (21.13%)	
**3-5 times/week**	46070 (21.78%)	
**times/week**	61442 (29.05%)	

Continuous variables are represented as the mean value with standard deviation in parentheses, except for HbA1c, Triglycerides and eGFR, which are represented as median with interquartile range in parenthesis because of positively skewed distributions. Dichotomous variables are represented as number of patients with percentage in parentheses.
BMI = body mass index, BP = blood pressure, eGFR = estimated glomerular filtration rate, HbA1c = glycated hemoglobin, HDL = high density lipoprotein, LDL = low density lipoprotein, n = number of patients.

Supplementary Table 3 HR comparing diabetics to controls adjusted for socioecominic variables for main anlaysis and excluding the first 1, 2 and 3 years of follow up

Tumour type	analysis using all follow up	1 year landmark analysis	2 years landmark analysis	3 years landmark analysis
ALLCANCER	1.10[1.09, 1.12]; p=<.0001	1.08[1.07, 1.10]; p=<.0001	1.08[1.06, 1.09]; p=<.0001	1.07[1.05, 1.08]; p=<.0001
BLADDER	1.20[1.15, 1.25]; p=<.0001	1.18[1.13, 1.24]; p=<.0001	1.17[1.11, 1.23]; p=<.0001	1.18[1.12, 1.25]; p=<.0001
BONE	1.21[0.83, 1.76]; p=0.3205	1.17[0.77, 1.80]; p=0.4634	1.34[0.84, 2.12]; p=0.2145	1.51[0.92, 2.49]; p=0.1059
BRAIN	1.14[1.05, 1.23]; p=0.0015	1.12[1.03, 1.22]; p=0.0094	1.09[1.00, 1.20]; p=0.0629	1.07[0.96, 1.19]; p=0.2326
BREAST	1.05[1.01, 1.09]; p=0.0093	1.04[1.00, 1.08]; p=0.0683	1.03[0.99, 1.08]; p=0.1437	1.00[0.96, 1.05]; p=0.9017
CERVIX	1.14[0.97, 1.34]; p=0.1094	1.10[0.93, 1.32]; p=0.2677	1.10[0.90, 1.33]; p=0.3651	1.11[0.89, 1.38]; p=0.3539
COLON	1.25[1.21, 1.30]; p=<.0001	1.23[1.19, 1.28]; p=<.0001	1.24[1.19, 1.29]; p=<.0001	1.24[1.19, 1.30]; p=<.0001
COLORECT	1.20[1.16, 1.23]; p=<.0001	1.19[1.16, 1.23]; p=<.0001	1.20[1.16, 1.24]; p=<.0001	1.20[1.16, 1.24]; p=<.0001
CORPUS	1.78[1.68, 1.88]; p=<.0001	1.75[1.64, 1.86]; p=<.0001	1.74[1.62, 1.87]; p=<.0001	1.73[1.60, 1.87]; p=<.0001
ESOPHAGUS	1.18[1.07, 1.31]; p=0.0014	1.20[1.07, 1.34]; p=0.0012	1.22[1.09, 1.38]; p=0.0009	1.30[1.14, 1.48]; p=<.0001
EYE	1.20[0.97, 1.49]; p=0.0911	1.16[0.92, 1.47]; p=0.2138	1.19[0.92, 1.54]; p=0.1803	1.11[0.82, 1.49]; p=0.5089
GALL	1.32[1.13, 1.54]; p=0.0005	1.32[1.12, 1.57]; p=0.0011	1.28[1.06, 1.54]; p=0.0106	1.26[1.02, 1.55]; p=0.0301
HODGKIN	1.19[0.93, 1.52]; p=0.1702	1.22[0.94, 1.58]; p=0.1371	1.29[0.97, 1.72]; p=0.0773	1.36[0.99, 1.85]; p=0.0544
KIDNEY	1.45[1.36, 1.54]; p=<.0001	1.43[1.34, 1.53]; p=<.0001	1.42[1.31, 1.52]; p=<.0001	1.40[1.29, 1.53]; p=<.0001
LARYNX	0.80[0.67, 0.96]; p=0.0159	0.69[0.56, 0.85]; p=0.0005	0.70[0.56, 0.88]; p=0.0019	0.69[0.54, 0.89]; p=0.0042
LEUKEMIA	1.15[1.04, 1.26]; p=0.0048	1.14[1.03, 1.27]; p=0.0134	1.17[1.04, 1.31]; p=0.0065	1.14[1.01, 1.29]; p=0.0377
LIP	1.27[1.06, 1.52]; p=0.0082	1.33[1.10, 1.61]; p=0.0028	1.37[1.12, 1.68]; p=0.0022	1.43[1.15, 1.79]; p=0.0016
LIVER	3.31[3.07, 3.58]; p=<.0001	3.44[3.16, 3.73]; p=<.0001	3.59[3.29, 3.93]; p=<.0001	3.80[3.44, 4.20]; p=<.0001
LUNG	1.01[0.97, 1.05]; p=0.5391	0.99[0.95, 1.03]; p=0.6562	1.00[0.96, 1.05]; p=0.9453	1.00[0.95, 1.05]; p=0.9102
MELANOMA	1.03[0.98, 1.08]; p=0.2987	1.01[0.95, 1.07]; p=0.8388	0.99[0.93, 1.06]; p=0.8314	1.00[0.93, 1.07]; p=0.9468
MOUTH	1.04[0.88, 1.22]; p=0.6401	1.07[0.89, 1.27]; p=0.4837	1.09[0.90, 1.33]; p=0.3567	1.15[0.93, 1.41]; p=0.1871
MULTMYELOM	0.93[0.84, 1.02]; p=0.1088	0.93[0.84, 1.03]; p=0.1709	0.95[0.85, 1.06]; p=0.3364	0.95[0.84, 1.07]; p=0.3766
NONHODGKIN	0.96[0.91, 1.02]; p=0.2190	0.96[0.90, 1.02]; p=0.1887	0.96[0.89, 1.03]; p=0.2274	0.95[0.88, 1.03]; p=0.2028
NOSE	1.06[0.79, 1.41]; p=0.7183	1.10[0.80, 1.49]; p=0.5653	1.01[0.72, 1.42]; p=0.9566	1.03[0.70, 1.52]; p=0.8829
ORALCAVITY_ALL	1.02[0.94, 1.10]; p=0.7039	1.05[0.97, 1.15]; p=0.2460	1.05[0.95, 1.15]; p=0.3633	1.04[0.94, 1.16]; p=0.4635
OTHFEMGEN	1.54[1.33, 1.78]; p=<.0001	1.56[1.33, 1.83]; p=<.0001	1.57[1.32, 1.88]; p=<.0001	1.61[1.32, 1.96]; p=<.0001
OTHLEUKEMIA	1.15[1.04, 1.27]; p=0.0048	1.14[1.03, 1.27]; p=0.0124	1.17[1.04, 1.31]; p=0.0075	1.16[1.02, 1.32]; p=0.0216
OTHUTERUS	1.65[1.33, 2.03]; p=<.0001	1.60[1.27, 2.02]; p=<.0001	1.56[1.20, 2.02]; p=0.0008	1.60[1.21, 2.13]; p=0.0011
OVARY	0.92[0.83, 1.02]; p=0.1158	0.92[0.82, 1.03]; p=0.1429	0.89[0.78, 1.01]; p=0.0796	0.86[0.75, 1.00]; p=0.0478
PANCREAS	2.19[2.06, 2.32]; p=<.0001	1.95[1.82, 2.09]; p=<.0001	1.82[1.69, 1.97]; p=<.0001	1.74[1.60, 1.90]; p=<.0001
PENIS	1.56[1.27, 1.91]; p=<.0001	1.55[1.24, 1.93]; p=<.0001	1.55[1.22, 1.98]; p=0.0004	1.61[1.23, 2.11]; p=0.0005
PHARYNX	0.94[0.81, 1.09]; p=0.4028	0.94[0.80, 1.11]; p=0.4917	0.90[0.75, 1.08]; p=0.2451	0.87[0.70, 1.07]; p=0.1783
PLEURA	0.80[0.65, 0.98]; p=0.0351	0.84[0.67, 1.05]; p=0.1201	0.84[0.66, 1.07]; p=0.1563	0.96[0.74, 1.24]; p=0.7337
PROSTATE	0.82[0.80, 0.83]; p=<.0001	0.79[0.77, 0.81]; p=<.0001	0.78[0.76, 0.80]; p=<.0001	0.77[0.75, 0.80]; p=<.0001
RECTAL	1.11[1.06, 1.16]; p=<.0001	1.12[1.06, 1.18]; p=<.0001	1.12[1.06, 1.19]; p=<.0001	1.12[1.06, 1.20]; p=0.0003
SALIVARY	0.88[0.67, 1.15]; p=0.3386	0.87[0.65, 1.16]; p=0.3308	0.75[0.54, 1.05]; p=0.0919	0.85[0.60, 1.21]; p=0.3708
SMALLINTEST	1.10[0.95, 1.27]; p=0.2091	1.09[0.93, 1.27]; p=0.2905	1.04[0.87, 1.24]; p=0.6623	1.02[0.84, 1.24]; p=0.8593
SOFTTISSUE	1.04[0.90, 1.21]; p=0.5581	1.00[0.85, 1.17]; p=0.9736	1.03[0.87, 1.23]; p=0.7091	0.98[0.81, 1.20]; p=0.8562
STOMACH	1.21[1.13, 1.30]; p=<.0001	1.21[1.12, 1.30]; p=<.0001	1.15[1.06, 1.26]; p=0.0013	1.14[1.03, 1.25]; p=0.0097
TESTIS	0.60[0.38, 0.95]; p=0.0293	0.57[0.35, 0.94]; p=0.0290	0.48[0.26, 0.90]; p=0.0218	

Tumour type	analysis using all follow up	1 year landmark analysis	2 years landmark analysis	3 years landmark analysis
THYROID	1.16[1.00, 1.35]; p=0.0492	1.20[1.02, 1.41]; p=0.0290	1.15[0.96, 1.38]; p=0.1194	1.13[0.92, 1.38]; p=0.2539
TONGUE	0.97[0.81, 1.15]; p=0.6967	1.06[0.88, 1.28]; p=0.5177	1.12[0.91, 1.37]; p=0.2875	0.97[0.76, 1.23]; p=0.7941

Supplementary Table 4 Hazard ratios in current study for most strongly associated cancers in type 2 diabetes vs. Hazard ratios associated with 5 unit BMI difference shown study in a mainly white European cohort

Cancer	HR in current study	HR associated with 5 unit BMI difference
**Liver**	3.31 (3.07–3.58)	1.26 (1.14–1.40)
**Pancreas**	2.19 (2.06−2.32)	1.11 (1.03–1.19)
**Corpus uteri**	1.78 (1.68−1.88)	1.63 (1.55–1.71)
**Gallbladder**	1.32 (1.13−1.54)	1.50 (1.21–1.85)
**Kidney**	1.45 (1.36−1.54)	1.25 (1.13–1.38)
**Penis**	1.56 (1.27−1.91)	-[a]
**Stomach**	1.21 (1.13−1.30)	1.08 (1.00–1.18)
**Bladder**	1.20 (1.15−1.25)	1.05 (0.99–1.12)
**Colorectal**	1.20 (1.16−1.23)	1.11 (1.07–1.15)[b]

HR associated with 5 unit BMI difference taken from: Body-mass index and risk of 22 specific cancers: a population-based cohort study of 5.24 million UK adults. Krishnan Bhaskaran, Ian Douglas, Harriet Forbes, Isabel dos-Santos-Silva, David A Leon, Liam Smeeth Lancet 2014; 384: 755–65 http://dx.doi.org/10.1016/S0140-6736(14)60892-8

[a] Data not available

[b] Colon cancer only

Supplementary Figure 1 Results for all cancer sites

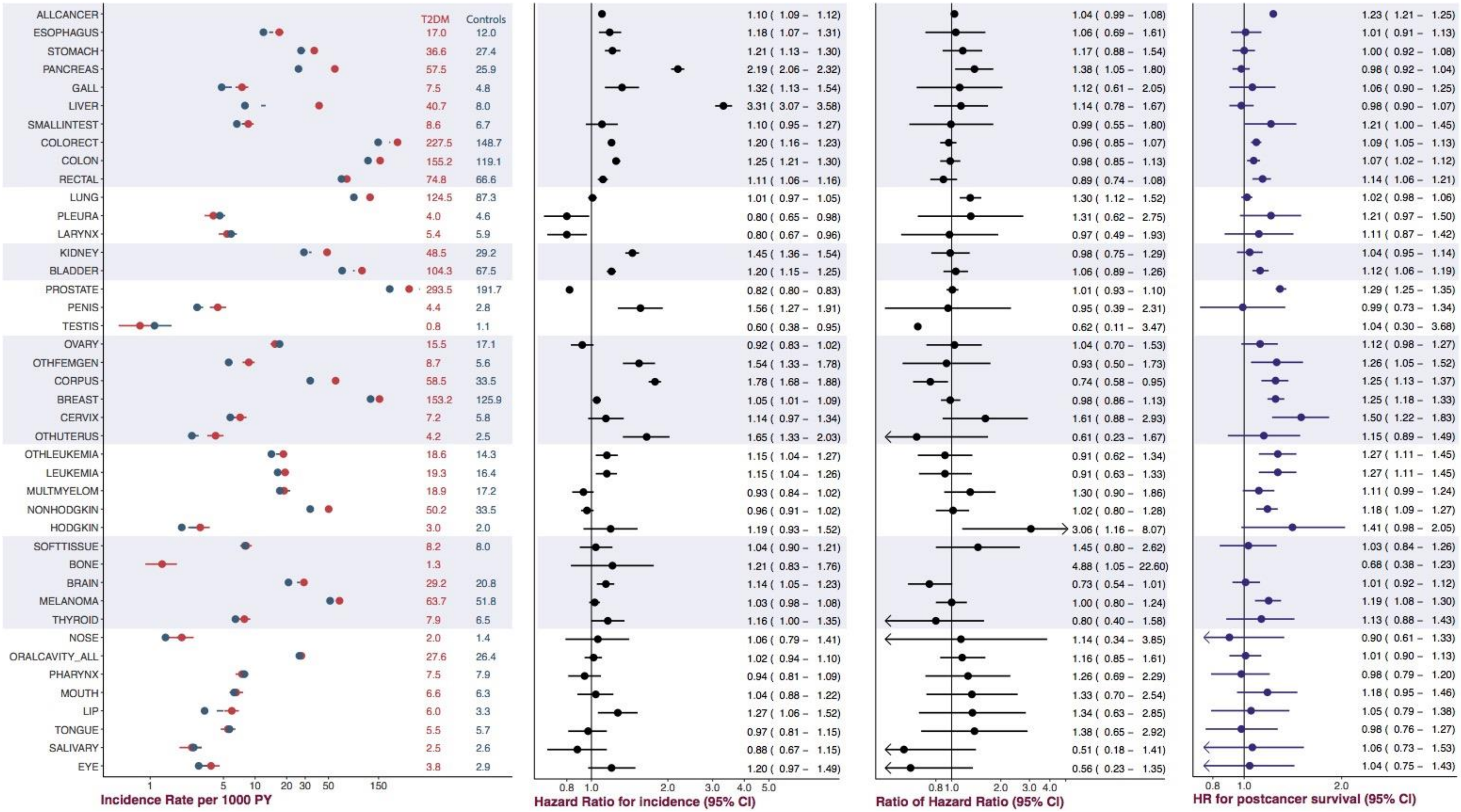

Supplementary Figure 2 Relative importance of risk factors (R^2) estimated with the Heller model

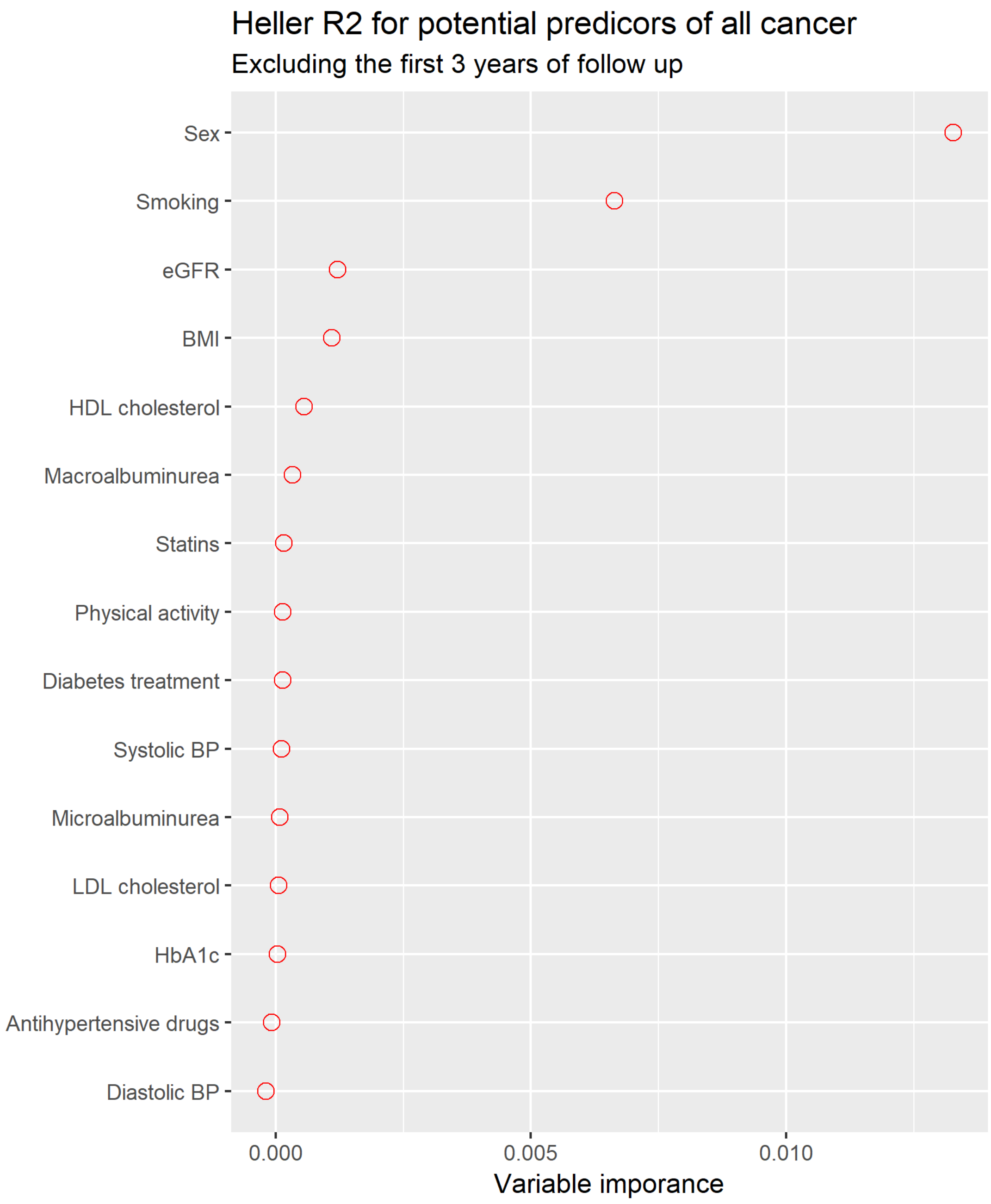

Supplementary Figure 3 Relative importance of risk factors (R^2) estimated with the Heller model in male non-smokers

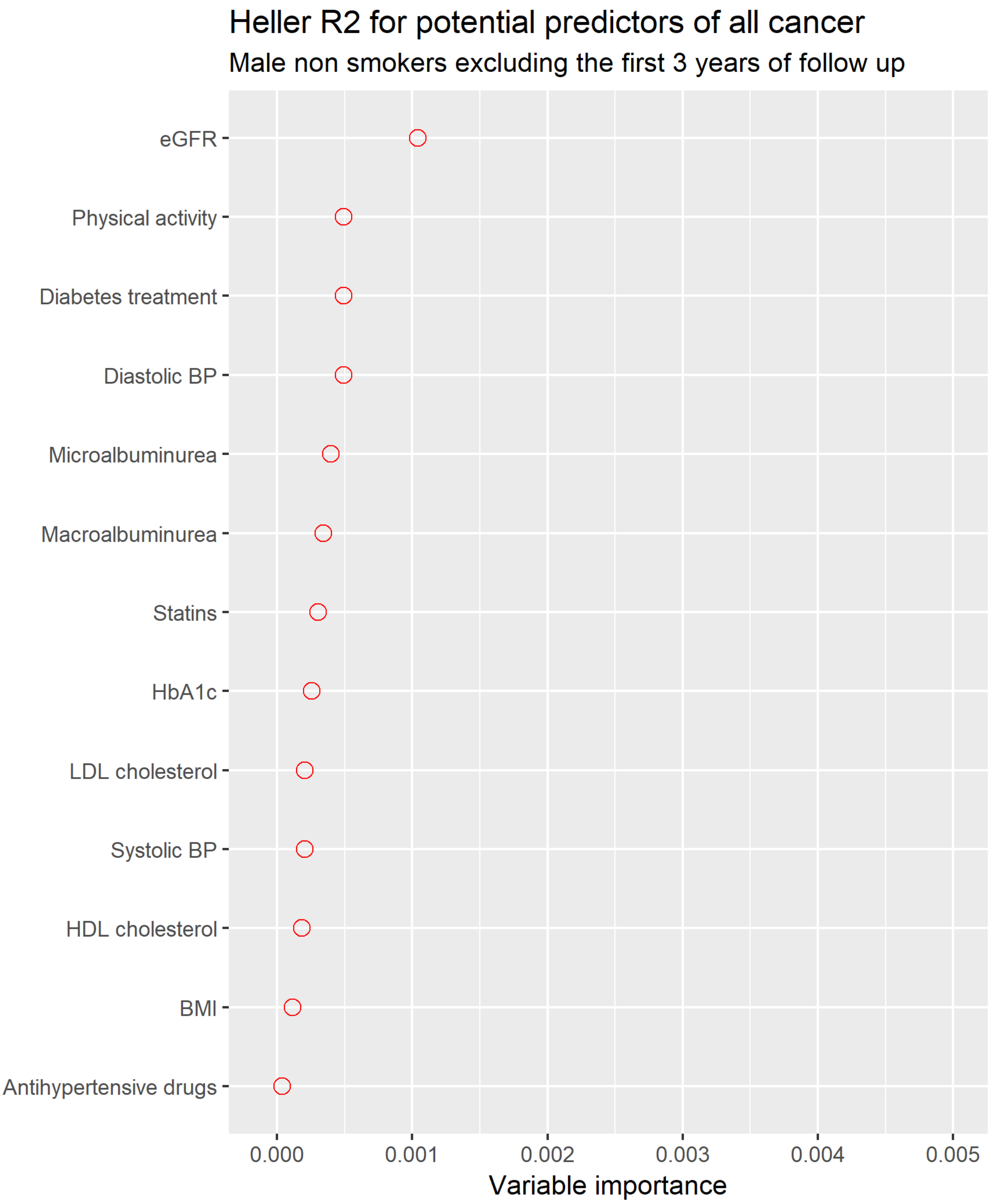

Supplementary Figure 4 Relative importance of risk factors (R^2) estimated with the Heller model in female non-smokers

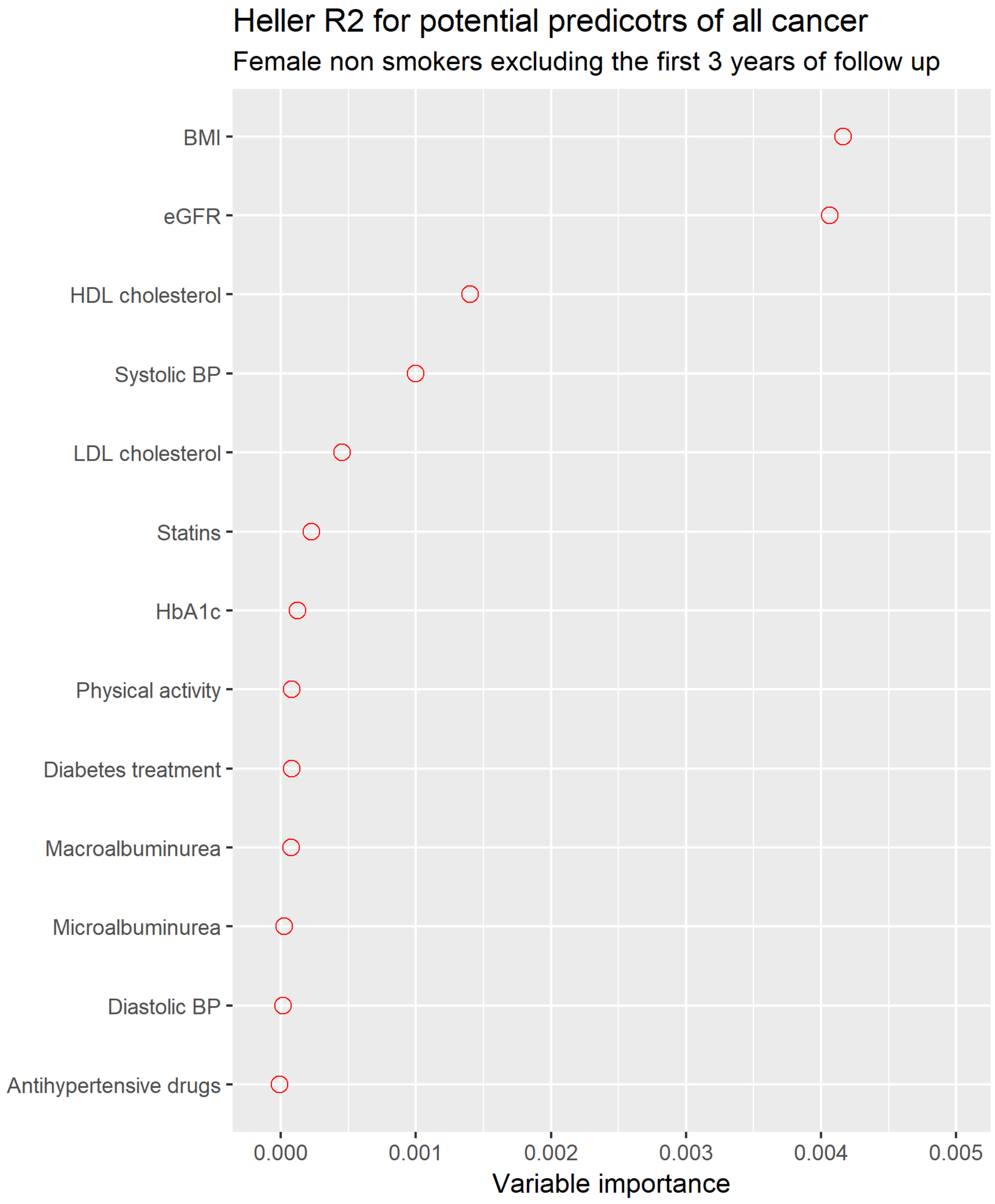

Supplementary Figure 5 Relative importance of risk factors (R^2) estimated with the Heller model, including diabetes duration

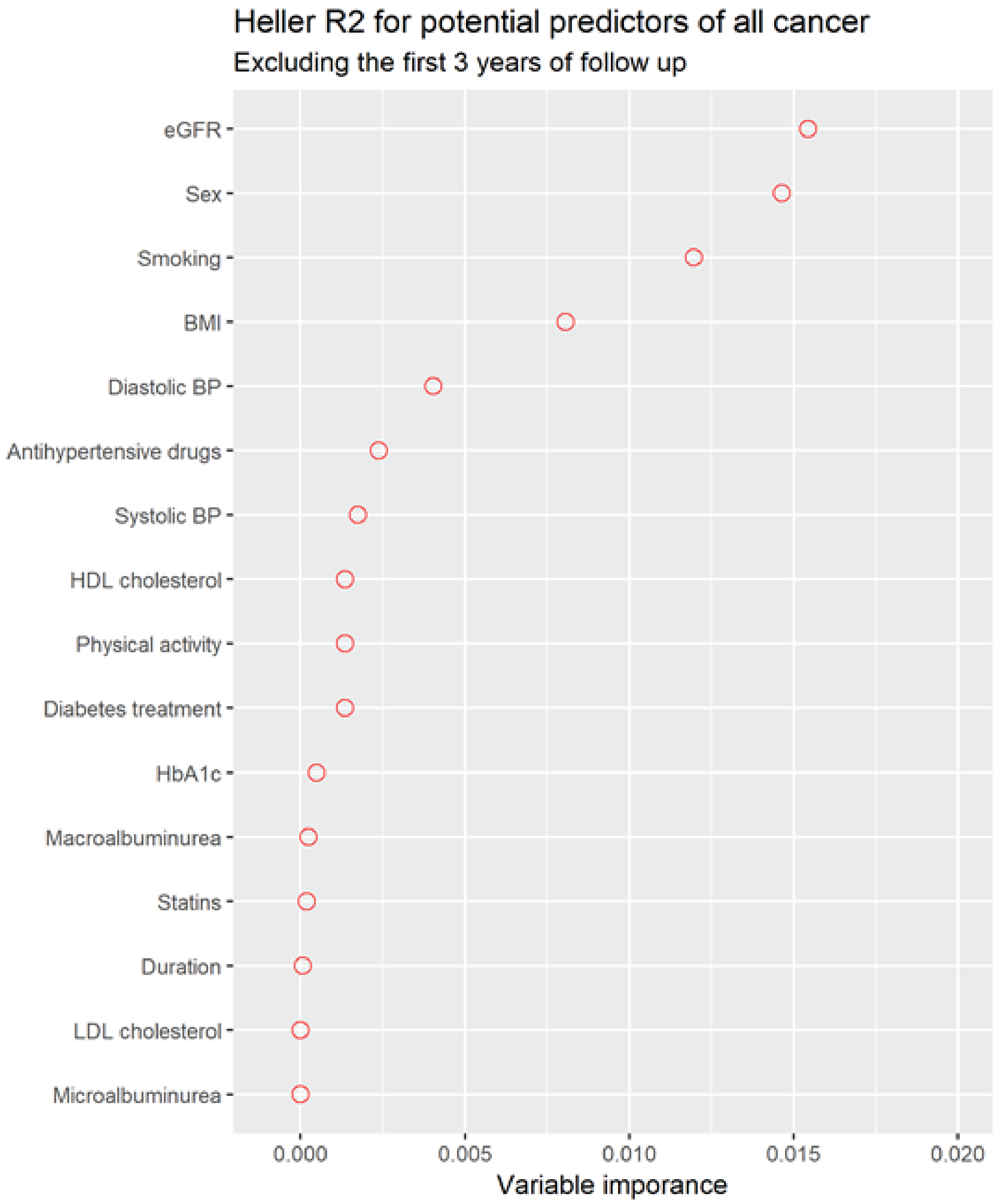

Supplementary Figure 6 Relative importance of risk factors (R^2) estimated with the Heller model in male non-smokers, including diabetes duration

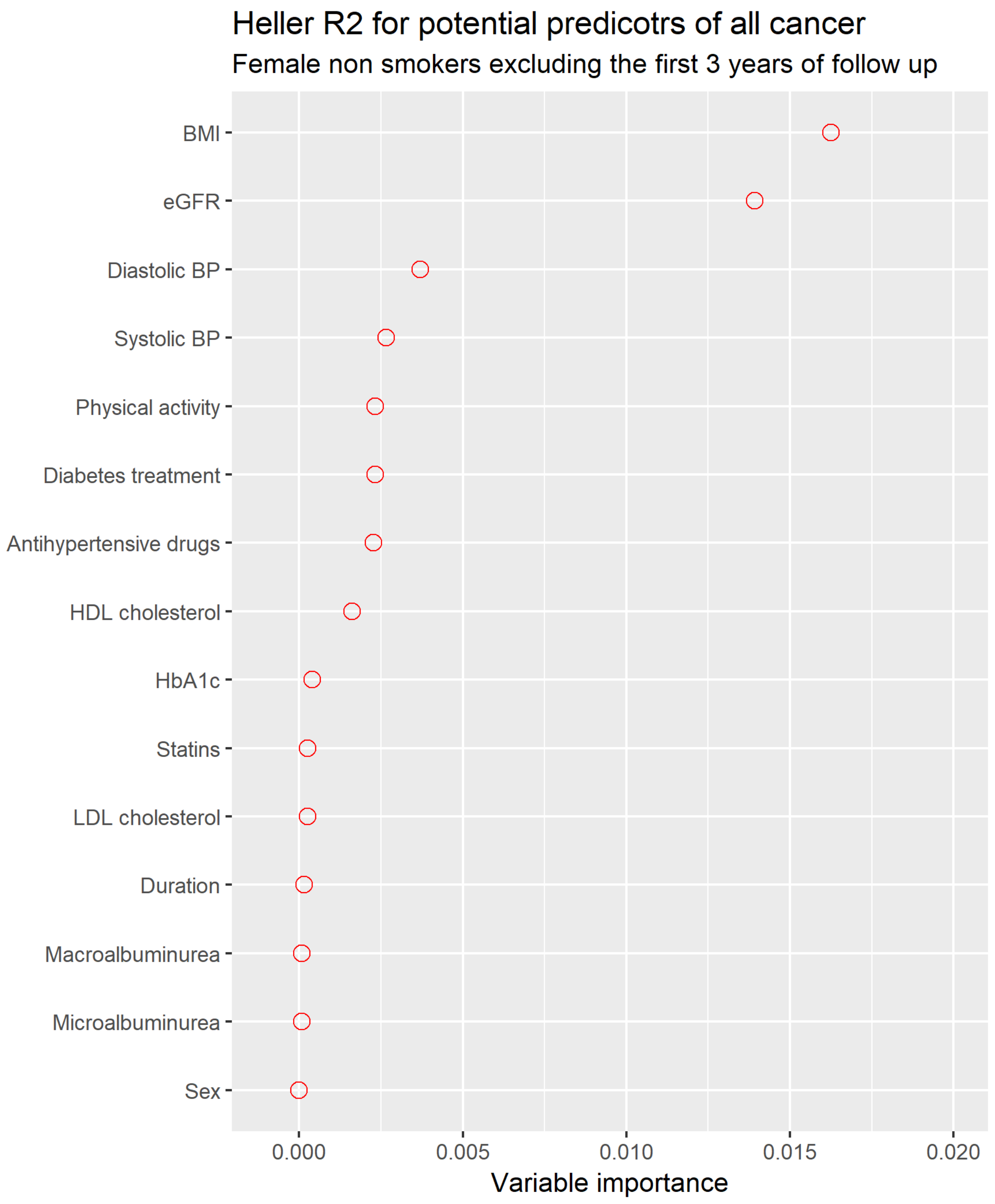

Supplementary Figure 7 Relative importance of risk factors (R^2) estimated with the Heller model in female non-smokers, including diabetes duration

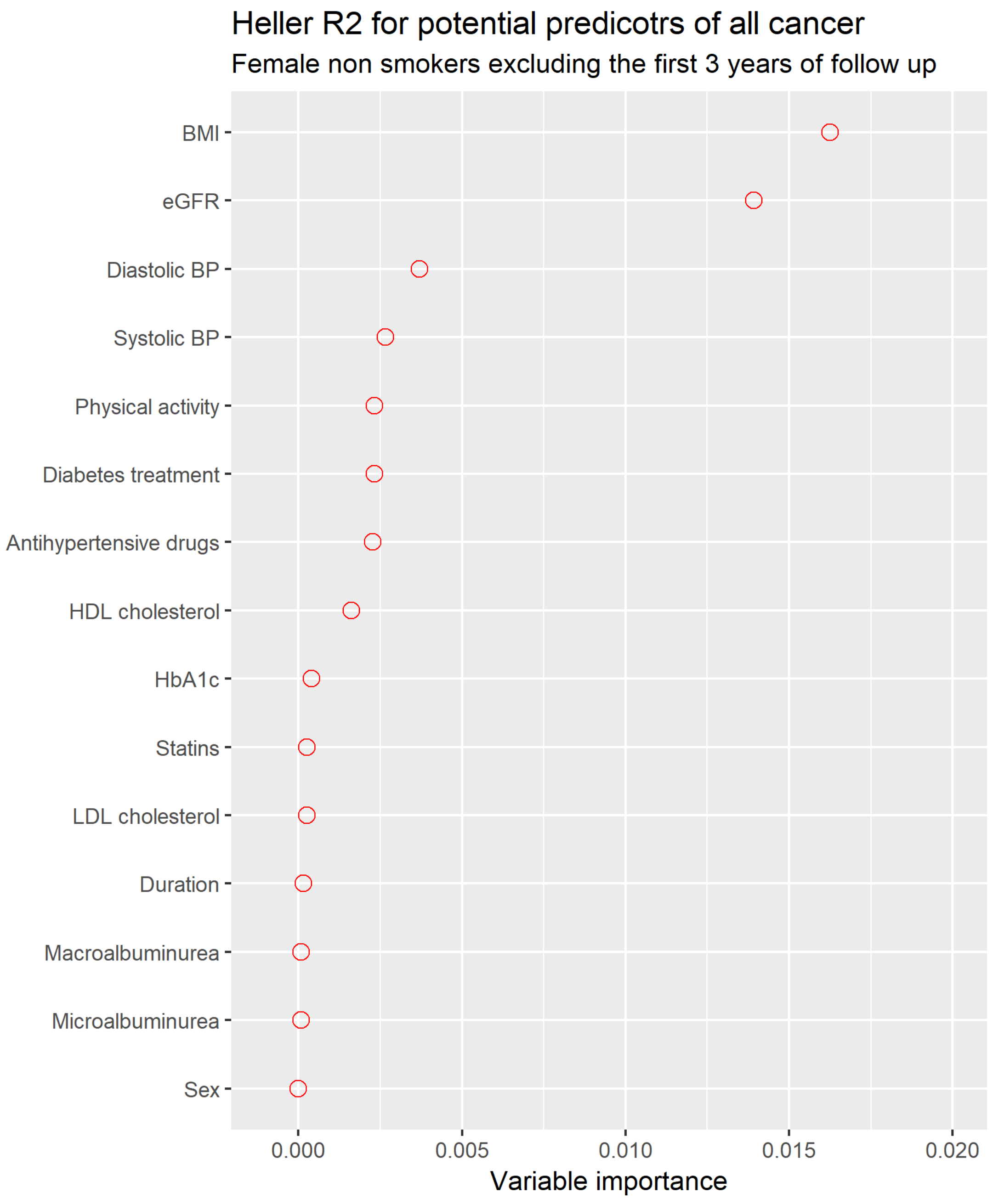

Supplementary Figure 8

Yearly incidence of pancreatic cancer per 10000 person years by time from diabetes diagnosis

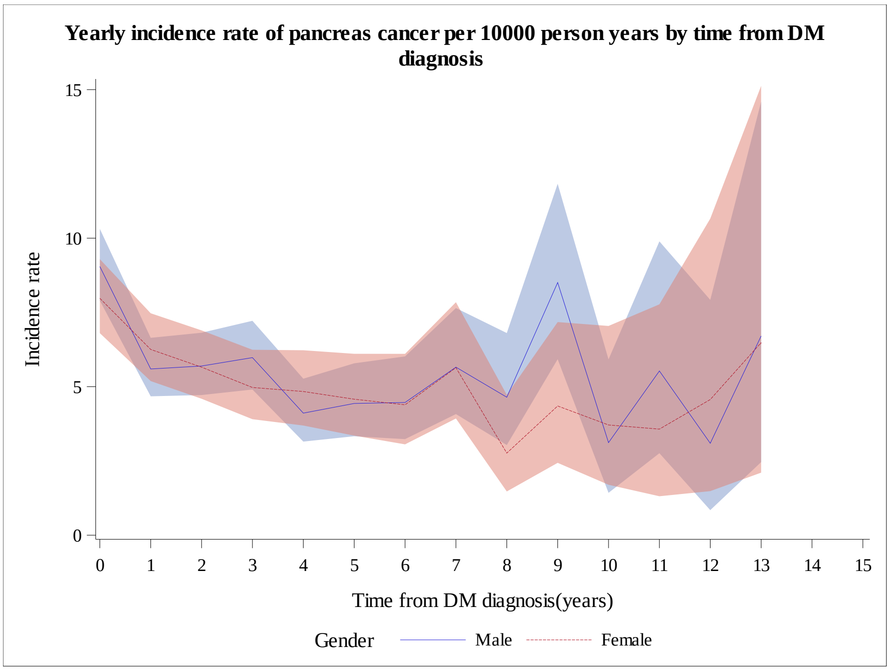

Supplementary Figure 9

Yearly incidence of all cancers per 10000 person years by time from diabetes diagnosis

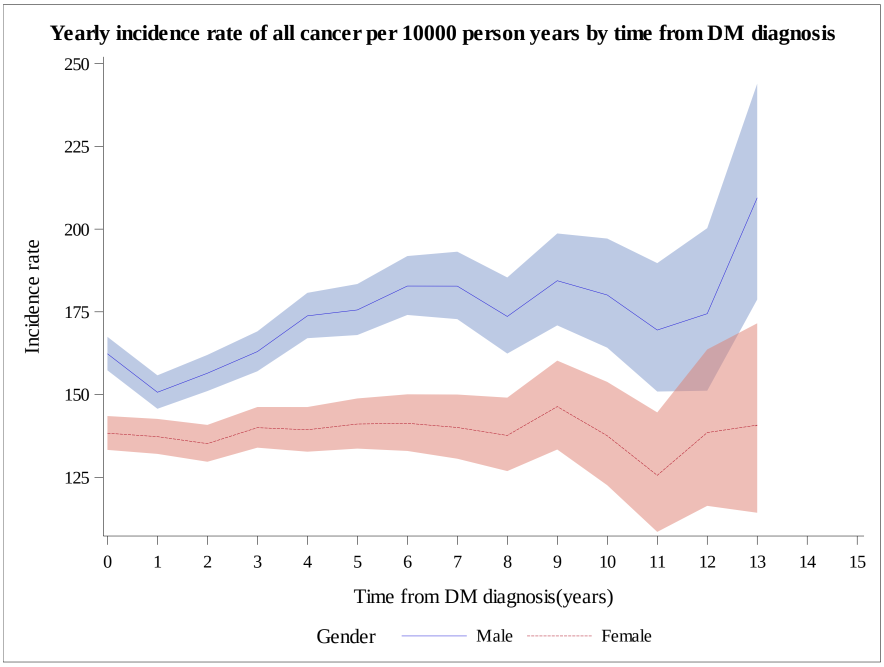

Supplement: Supplementary file 1 — Supplementary Information. [file 41598_2020_73668_MOESM1_ESM.pdf]
